# Managing biological invasions in urban environments with the acceptance sampling approach

**DOI:** 10.1371/journal.pone.0220687

**Published:** 2019-08-23

**Authors:** Denys Yemshanov, Robert G. Haight, Cuicui Chen, Ning Liu, Christian J. K. MacQuarrie, Frank H. Koch, Robert Venette, Krista Ryall

**Affiliations:** 1 Natural Resources Canada, Canadian Forest Service, Great Lakes Forestry Centre,Sault Ste. Marie, ON, Canada; 2 USDA Forest Service, Northern Research Station, St. Paul, MN, United States of America; 3 University at Albany, State University of New York, Albany, NY, United States of America; 4 USDA Forest Service, Southern Research Station, Eastern Forest Environmental Threat Assessment Center, Research Triangle Park, NC, United States of America; 5 USDA Forest Service, Northern Research Station, St. Paul, MN, United States of America; Ecole des Mines d'Ales, FRANCE

## Abstract

Detections of invasive species outbreaks are often followed by the removal of susceptible host organisms in order to slow the spread of the invading pest population. We propose the acceptance sampling approach for detection and optional removal of susceptible host trees to manage an outbreak of the emerald ash borer (EAB), a highly destructive forest pest, in Winnipeg, Canada. We compare the strategy with two common delimiting survey techniques that do not consider follow-up management actions such as host removal. Our results show that the management objective influences the survey strategy. The survey-only strategies maximized the capacity to detect new infestations and prioritized sites with high likelihood of being invaded. Comparatively, the surveys with subsequent host removal actions allocated most of the budget to sites where complete host removal would minimize the pest’s ability to spread to uninvaded locations. Uncertainty about the pest’s spread causes the host removal measures to cover a larger area in a uniform spatial pattern and extend to farther distances from already infested sites. If a decision maker is ambiguity-averse and strives to avoid the worst-case damages from the invasion, the optimal strategy is to survey more sites with high host densities and remove trees from sites at farther distances, where EAB arrivals may be uncertain, but could cause significant damage if not detected quickly. Accounting for the uncertainty about spread helps develop a more robust pest management strategy. The approach is generalizable and can support management programs for new pest incursions.

## Introduction

When an invasive species is detected in a novel environment, rapid response measures provide a way to impede the invader’s advance and limit its potential impacts [1–5]. For example, in management programs for wood-boring invasive insects in urban forests, successful detections (e.g., via tree inspections) may be followed by the removal of host trees that are infested or susceptible to attack. In such cases, host removal helps slow the expansion of the invader’s population into previously uninvaded areas and, if implemented at early stages of the invasion, may lead to successful eradication [6,7].

However, host removal is expensive, especially when compared to the cost of surveillance on a per-unit basis. Limited funding may render the host removal measures ineffective if too few trees are removed, while managers may attempt to balance the surveillance and removal efforts to maximize their joint effectiveness. Optimization-based models have been used to identify combinations of survey and strategic removal of selected trees to serve overall management objectives [8–11]. These models can be quite sophisticated in terms of computational scope. Recently, some authors have proposed the use of optimization-based strategies to determine how to allocate funds for surveys and control actions when managing invasions in geographical space [5,9,12–17] and through time [18,19].

To be effective, surveys need to cover large areas in order to uncover the full spatial extent of an invasion. For example, one strategy in delimiting surveys is to attempt to maximize the number of sites (or total area) with successful detections of an invader of interest (i.e., to find all the sites where the invader has established). However, the success of this particular strategy will be influenced by the fact that the manager knows that any successful detection will lead to the application of a control measure. Thus, a portion of funds has to be set aside for those control measures, which either reduces the potential number of sites that can be surveyed or the surveys must be implemented at lower sampling rates. Reducing the surveyed area or sampling at a lower rate may lead to fewer detections, thus creating a trade-off between the relative amounts of funds that can be spent on surveys or control actions.

In practice, resource allocation decisions for surveillance and control are even more challenging because knowledge about the true extent of an invasion is always lacking. This uncertainty forces managers to rely on vague expectations of where and when an invader might establish when planning detection surveys. If these expectations ignore the uncertainty about the invader’s future spread, the surveys based on these expectations will underestimate the true extent of the invasion and thus create an optimistic perception of the efficacy of any control efforts that follow the detections. Furthermore, any other management actions based on these expectations may lead to failed detections and delays with eradication decisions, which, in turn, would amplify the potential damage from an invasion.

Statistical quality control methods such as acceptance sampling [20] provide effective means to account for potential negative outcomes of invasive species management decisions under uncertainty [21]. The acceptance sampling technique allows inspectors to accept or reject a lot (i.e., a group of items) based on information obtained from a sample of inspected items in the lot [22]. Acceptance sampling plays an important role in public health and food safety control programs [23–27] and controlling harmful pest entries with agricultural or ornamental plant imports [21,28,29].

Chen et al. [21] and Yemshanov et al. [30] applied acceptance sampling to the problems of invasive pest detection in plant imports and via geographical delimiting surveys. Here, we extend the acceptance sampling approach proposed in [21] and [30] to a geographical pest management problem with optional control measures, namely, the removal of infested and susceptible host plants (trees) after detection.

We divide the survey area into a spatial grid of survey sites and consider each site analogous to a lot with items that can be selected for inspections. The items in this case are the suitable host trees in each site, which can be inspected for visible signs of infestation. A sample of these trees is inspected and if one or more trees is found to be infested, the site is declared as invaded. A decision maker may choose after detection to remove a portion of trees from the sampled tree population and from the rest of the tree population in the invaded site.

We find optimal allocations of survey and tree removal actions (at different budget levels) that minimize the expected number of infested trees in the area after completion of these actions. We compare two optimal surveillance strategies. The first strategy includes optional tree removal measures which may follow the detection of the infested trees. The second strategy follows the approach presented in [30], which does not assume any tree removal or control actions after a detection and evaluates two alternative survey objectives (described as problem 1 and 2 in [30]). The first survey objective maximizes the expected number of sites with positive detections in the area, while the second objective minimizes the expected number of infested trees in sites where surveys failed to detect the infested trees or where no survey was conducted. We propose a linear programming model that allocates survey and tree removal measures when the likelihoods of pest introduction and spread in the managed area are uncertain. We also examine how a decision-maker’s perception of uncertainty, such as an ambiguity-averse perspective that aspires to avoid the worst-case outcomes of management actions, may influence the tree removal choices. We apply our approach to a case study of the emerald ash borer (EAB), *Agrilus planipennis* Fairmaire (Coleoptera: Buprestidae), in Winnipeg, Canada. EAB is an invasive forest insect that was discovered in Winnipeg in December 2017 [31]. The EAB is native to eastern Asia and poses a major threat to North American ash (*Fraxinus*) trees. Since its initial introduction in Michigan, it has caused catastrophic damage in the eastern United States and Canada, with estimated tree replacement costs of over US $12B and lost property values of over $3.8B [32–37]. Currently, the EAB infestation in Winnipeg is at its early stage (with a significant number of detections made via branch sampling in asymptomatic trees) and a higher proportion of the population is estimated to have a longer, two-year life cycle compared to populations where the shorter one-year lifecycle of EAB predominate (e.g., southern Ontario and the eastern U.S.). Selecting and removing trees at the early stage of an infestation, when done in conjunction with branch sampling that enables early detection of EAB in asymptomatic trees, could reduce the incidence of new infestation nuclei in an area and buy time for cost-effective host removal actions before the infestation spreads uncontrollably and becomes too costly to eradicate.

## Materials and methods

Our spatial optimization model for surveillance and host removal depicts the uncertain likelihoods of invasion with a set of probabilistic scenarios (see Table 1 for symbol definitions). Consider an area of *J* sites that may be invaded by a pest. Each site *j*, *j ϵ J*, has *N*_*j*_ host trees that may be infested. The manager chooses an inspection intensity *m*, *m ϵ M*, for each site *j*, representing a sample size of *n*_*jm*_ trees to inspect for infestation. One of the inspection intensities assumes no inspections (i.e., *n*_*jm*_ = 0). For each site and inspection intensity, we define a binary decision variable *x*_*jm*_, where *x*_*jm*_ = 1 if inspection intensity *m* is selected for site *j* and *x*_*jm*_ = 0 otherwise. In our case, inspection intensity defines the number of trees *m* inspected at a site *j*. Only one inspection intensity level is allowed for each site. We define *e*_*j*_ as the detection rate, which is the probability that an inspection of a tree finds an infestation if it is present. Inspection of a tree at a site *j* has cost *g*_*j*_ and the total cost is constrained by an upper budget limit *B*.

**Table 1:** Summary of the model parameters and decision variables.

Symbol	Parameter / variable name	Description
**Sets:**		
*J*	Potential 1-km^2^ survey sites in the managed area *j*	*j ϵ J*, *J =* 472
*S*	Infestation scenarios *s*. Each scenario *s* defines a plausible pattern of infestation likelihoods, γ_*js*_ in the managed area *J*	*s ϵ S*. *S =* 2000
*M*	Survey sampling levels *m* for a site *j*. Each level *m* specifies sampling *n*_*jm*_ trees at a site *j*	*m ϵ M*
**Parameters**		
*B*	Survey budget constraint	*B* > 0
*N*_*j*_	Number of host trees at a site *j*	*N*_*j*_ ≥ 0
γ_*js*_	Likelihood of that a tree is infested in a site *j* in a scenario *s* (γ_*js*_*N*_*j*_−expected number of infested trees at a site *j* in a scenario *s*);	γ_*js*_ *ϵ* [0; 1]
*e*_*j*_	Probability of that inspections of an infested tree at a site *j* detect the signs of infestation	*e*_*j*_ *ϵ* [0; 1]
*P*_*jms*_	Probability of that inspections, at a sampling level *m*, fail to detect the infested tree(s) at a site *j* in a scenario *s*.	*P*_*jms*_ *ϵ* [0;1]
*g*_*j*_	Cost of surveying a tree at a site *j*	*g*_*j*_ > 0
*c*_*j*_	Cost of removing a tree at a site *j*	*c*_*j*_ *>* 0
*n*_*jm*_	Number of trees inspected at a site *j* at a survey sampling level *m*. The sampling level *n*_*jm*_ = 0 assumes no survey at a site *j*;	*n*_*jm*_ *ϵ* [0; *N*_*j*_]
*E*_*jms*_	Expected number of infested trees in a site *j* conditional on an inspection of *n*_*jm*_ trees at a sampling level *m* does not find the infested trees in a scenario *s*	*E*_*jms*_ *ϵ* [0; γ_*js*_*N*_*j*_]
*F*_*jms*_	Expected number of infested trees in a site *j* conditional on an inspection of *n*_*jm*_ trees at a sampling level *m* detects one or more infested trees in a scenario *s*	*F*_*jms*_ *ϵ* [0; γ_*js*_*N*_*j*_]
*β*_*js*_	Normalizing factor that conditions the number of infested trees in a sampled tree population *n*_*jm*_ on failing to detect the infested trees in a sample of *n*_*jm*_ trees in a scenario *s*	
*P*	Probability of that inspections fail to detect one or more infested trees at a survey site	*P ϵ* [0;1]
*D*_1_	Expected number of infested trees at a surveyed site among those that were not inspected,	*D*_1_ *ϵ* [0; γ_*js*_*N*_*j*_]
*D*_2_	Expected number of infested trees among those inspected, conditional on the fact that the survey fails to find the signs of infestation	*D*_2_ *ϵ* [0; γ_*js*_*N*_*j*_]
*D*_3_	Expected number of infested trees among those inspected, conditional on the fact that the survey fails finds the signs of infestation	*D*_3_ *ϵ* [0; γ_*js*_*N*_*j*_]
**Decision variables:**		
*x*_*jm*_	Binary selection of a survey at a site *j* at a sampling level *m* (i.e., inspecting *n*_*jm*_ trees)	*x*_*jm*_ *ϵ* {0,1}
*y*_1*jm*_	Proportion of trees removed from a sampled population of *n*_*jm*_ trees at a site *j*	*y*_1*jm*_ *ϵ* [0;1]
*y*_2*jm*_	Proportion of trees removed from an unsampled population of *N*_*j*_−*n*_*jm*_ trees at a site *j*	*y*_2*jm*_ *ϵ* [0;1]

Let γ_*j*_ be the infestation rate of trees in site *j*, which denotes the likelihood that a tree in site *j* is infested. The knowledge of the infestation rates γ_*j*_ for all sites *j ϵ J* is uncertain. Based on prior information about the infestation in the area and historical estimates of spread rates for other regions, we define *S* scenarios of infestation rates. Each scenario *s ϵ S* is a vector of infestation rates γ_*js*_, for all sites *j*, *j ϵ J*, where each element γ_*js*_ depicts the infestation rate at a given site *j*.

The manager chooses the number of trees to inspect in each survey site, *n*_*jm*_. If one or more of the trees in the sample of inspected trees is found infested, then the site is declared infested. We adapt the acceptance sampling formula from Chen et al. [21] to determine the expected number of infested trees, *E*_*jms*_, in a site *j* after an inspection of *n*_*m*_ trees fails to find the infestation in a scenario *s*, i.e.:

$$E_{jms}={\left(1-\gamma_{js}e_{j}\right)}^{n_{jm}}\left[\gamma_{js}(N_{j}-n_{jm})+\frac{1-e_{j}}{1-\gamma_{js}e_{j}}\gamma_{js}n_{jm}\right]=P\{D_{1}+D_{2}\}.$$ (1)

Term *P* denotes the probability that none of the inspected trees were found to be infested, *D*_*1*_ is the expected number of infested trees among those that were not inspected, and *D*_*2*_ is the expected number of infested trees among those inspected, conditional on the fact that the site was declared uninfested. Using terminology from Chen et al. [21], Eq (1) defines the *expected slippage* when failing to detect an infestation in a site *j*. Note that when no trees are inspected in site *j*, *n*_*jm*_ = 0 and *E*_*jms*_ = γ_*js*_*N*_*j*_, which is the expected number of infested trees in site *j*.

If one or more trees in an inspected sample *n*_*jm*_ in a scenario *s* are found to be infested, the site is declared infested and the decision maker may decide to remove a portion of host trees from the site. The manager may choose to remove a portion of trees from the sampled population, *y*_1*jm*_, in a site *j* in a scenario *s* and a portion of the remaining population of unsampled trees, *y*_2*jm*_. No trees are removed from sites that are declared uninfested (i.e., when the inspection of *n*_*jm*_ trees did not find signs of infestation) or sites that were not surveyed. The cost of removing a tree at a site *j* is *c*_*j*_. Tree removal decisions at a site *j* are not scenario-specific and apply to all *S* invasion scenarios because knowledge about the infestation rates at the survey sites is uncertain.

Our problem objective is to choose the inspection intensity and tree removal proportions for the sites in the managed area with positive detections that minimize the expected number of infested trees remaining in the landscape after tree removal. This problem objective is subject to an upper bound on the total survey and tree removal budget. First, we use Eq (1) to estimate the expected number of infested trees in the sites where the inspection of a sample of *n*_*jm*_ trees did not find the signs of infestation (i.e., the expected slippage when failing to detect an infestation). We then estimate the expected number of infested trees in the sites where the inspection of a sample of *n*_*jm*_ trees has detected the infestation as:

$$F_{jms}=[1-{\left(1-\gamma_{js}e_{j}\right)}^{n_{jm}}]\left[\gamma_{js}(N_{j}-n_{jm})+n_{jm}\gamma_{js}\frac{1-(1-e_{j}){\left(1-\gamma_{js}e_{j}\right)}^{n_{jm}-1}}{1-{\left(1-\gamma_{js}e_{j}\right)}^{n_{jm}}}\right]=(1-P)\{D_{1}+D_{3}\}.$$ (2)

Term 1 –*P* denotes the probability that the inspections find one or more infested trees in a population of sampled trees and *D*_3_ is the expected number of infested trees among those inspected conditional on that the site is declared infested.

Eqs (1) and (2) can be written as:

$$E_{jms}=\gamma_{js}(N_{j}-n_{jm}){\left(1-\gamma_{js}e_{j}\right)}^{n_{jm}}+n_{jm}\gamma_{js}(1-e_{j}){\left(1-\gamma_{js}e_{j}\right)}^{n_{jm}-1}$$ (3)

$$F_{jms}=\gamma_{js}(N_{j}-n_{jm})[1-{\left(1-\gamma_{js}e_{j}\right)}^{n_{jm}}]+n_{jm}\gamma_{js}[1-(1-e_{j}){\left(1-\gamma_{js}e_{j}\right)}^{n_{jm}-1}]$$ (4)

Let *P*_*jms*_ be the probability that the survey fails to detect one or more infested trees in a sample of trees *n*_*jm*_ in a scenario s:

$$P_{jms}={\left(1-\gamma_{js}e_{j}\right)}^{n_{jm}}$$ (5)

and *β*_*js*_ be the adjustment factor for the number of infested trees in the inspected sample of trees in Eq (1):

$$\beta_{js}=\frac{1-e_{j}}{1-\gamma_{js}e_{j}}.$$ (6)

Given the equality (1 –*e*_*j*_)(1 –γ_*js*_*e*_*j*_)^*njm*-1^ = *P*_*jms*_*β*_*js*_, Eqs (3) and (4) can be rewritten as:

$$E_{jms}=P_{jms}\gamma_{js}(N_{j}-n_{jm})+n_{jm}\gamma_{js}P_{jms}\beta_{js}$$ (7)

$$F_{jms}=\gamma_{js}(N_{j}-n_{jm})[1-P_{jms}]+n_{jm}\gamma_{js}[1-P_{jms}\beta_{js}].$$ (8)

The sum of the expected numbers of infested trees conditional on detecting and failing to detect the infestation is equal to the expected number of infested trees at a site *j*, i.e:

$$E_{jms}+F_{jms}=\gamma_{js}N_{j}.$$ (9)

We then formulate the objective function as minimizing the expected number of infested trees in a site *j* where the inspection of *n*_*jm*_ trees did not find an infestation or no survey occurred, *E*_*jms*_, and the expected number of infested trees after the inspection of *n*_*jm*_ trees has found one or more infested trees in a scenario *s* and tree removal may have occurred, *F*_*jms*_:

$$z=\min\frac{1}{S}\sum_{s=1}^{S}\sum_{j=1}^{J}\sum_{m=1}^{M}(x_{jm}E_{jms}+x_{jm}F_{jms}),$$ (10)

where *E*_*jms*_ is defined in Eq (7) and

$$F_{jms}=\gamma_{js}(N_{j}-n_{jm})[1-P_{jms}](1-y_{2jm})+n_{jm}\gamma_{js}[1-P_{jms}\beta_{js}](1-y_{1jm}).$$ (11)

Terms 1 –*y*_1*jm*_ and 1 –*y*_2*jm*_ denote the proportions of the remaining infested trees in a sample of *n*_*jm*_ inspected trees and the unsampled tree population *N*_*j*_—*n*_*jm*_, and terms *P*_*jms*_ and *β*_*js*_ are defined in Eqs (5) and (6).

Terms *x*_*jm*_*E*_*jms*_ and *x*_*jm*_*F*_*jms*_ in Eq (10) can be written as:

$$E_{jms}x_{jm}=x_{jm}P_{jms}\gamma_{js}(N_{j}-n_{jm})+x_{jm}n_{jm}\gamma_{js}P_{jms}\beta_{js}$$ (12)

and

$$F_{jms}x_{jm}=x_{jm}\gamma_{js}(N_{j}-n_{jm})[1-P_{jms}](1-y_{2jm})+x_{jm}n_{jm}\gamma_{js}[1-P_{jms}\beta_{js}](1-y_{1jms}).$$ (13)

The products of the binary decision variables *x*_*jm*_ and non-negative tree removal variables *y*_1*jm*_ and *y*_2*jm*_ in Eq (13) can be replaced with the variables *y*_1*jm*_ and *y*_2*jm*_ and the constraint (18), which ensures that *y*_1*jm*_ and *y*_2*jm*_ = 0 when *x*_*jm*_ = 0 or *n*_*jm*_ = 0, i.e.:

$$F_{jms}x_{jm}=\gamma_{js}(N_{j}-n_{jm})[1-P_{jms}](x_{jm}-y_{2jm})+n_{jm}\gamma_{js}[1-P_{jms}\beta_{js}](x_{jm}-y_{1jm}).$$ (14)

Term *x*_*jm*_(*E*_*jms*_ + *F*_*jms*_) can be written as:

$$\begin{array}{l} \gamma_{js}(N_{j}-n_{jm})[1-P_{jms}](x_{jm}-y_{2jm})+n_{jm}\gamma_{js}[1-P_{jms}\beta_{js}](x_{jm}-y_{1jm})+x_{jm}P_{jms}\gamma_{js}(N_{j}-n_{jm})+x_{jm}n_{jm}\gamma_{js}P_{jms}\beta_{js}=\\ =\gamma_{js}(N_{j}-n_{jm})(x_{jm}-y_{2jm}[1-P_{jms}])+\gamma_{js}n_{jm}(x_{jm}-y_{1jm}[1-P_{jms}\beta_{js}]) \end{array}$$ (15)

and the objective function equation after rearranging as:

$$z=\min\frac{1}{S}\sum_{s=1}^{S}\sum_{j=1}^{J}\sum_{m=1}^{M}\left[x_{jm}\gamma_{js}P_{jms}(N_{j}-n_{jm}[1-\beta_{js}])+\gamma_{js}[1-P_{jms}](N_{j}-n_{jm})(x_{jm}-y_{2jm})+\gamma_{js}n_{jm}[1-P_{jms}\beta_{js}](x_{jm}-y_{1jm})\right]$$ (16)

s.t.:

$$0\leq y_{1jm},y_{2jm}\leq 1\forall \;j\in J,m\in M,s\in S$$ (17)

$$y_{1jm},y_{2jm}\leq x_{jm}n_{jm}\;\forall \;j\in J,m\in M,s\in S,$$ (18)

assuming the lowest positive sample size *n*_*jm*_ = 1. Constraint (17) imposes lower and upper bounds on the proportions of trees removed from the sampled and unsampled populations, *y*_1*jm*_ and *y*_2*jm*_. Constraint (18) ensures that tree removal could only occur at sites that are surveyed (i.e., *y*_1*jm*_ and *y*_2*jm*_ = 0 when *n*_*jm*_ = 0) and also ensures that only one set of *y*_1*jm*_ and *y*_2*jm*_ variables out of *M* possible survey intensity levels *m* can be positive at a site *j* (i.e., *y*_1*jm*_, *y*_2*jm*_ = 0 when *x*_*jm*_ = 0).

As noted earlier, the budget constraint (19) limits the total number of inspected and removed trees by an upper bound in each infestation scenario:

$$\sum_{j=1}^{J}\sum_{m=1}^{M}\left[x_{jm}n_{jm}g_{j}+c_{j}(1-P_{jms})[(N_{j}-n_{jm})y_{2jm}+n_{jm}y_{1jm}]\right]\leq B\;\forall s\in S$$ (19)

where *n*_*jm*_*y*_1*jm*_ and (*N*_*j*_*−n*_*jm*_)*y*_2*jm*_ are the expected numbers of trees removed from the sampled and unsampled tree populations in site *j*.

Constraint (20) specifies that only one sample size *m* out of possible *M* sampling levels *n*_*jm*_ can be chosen for a survey implemented at a site *j*, i.e.:

$$\sum_{m=1}^{M}x_{jm}=1\;\forall \;j\in J.$$ (20)

### Surveys with host removal vs. survey-only strategies

We compared our survey strategy with optional host removal with two common delimiting survey strategies (as shown in Yemshanov et al. [30]). The first strategy (problem 1 hereafter) minimizes the expected number of sites with undetected infestations in area *J* across a set of infestation rate scenarios *S*, subject to the inspection budget constraint (22), i.e.:

$$z_{1}=\min\frac{1}{S}\sum_{s=1}^{S}\sum_{j=1}^{J}\sum_{m=1}^{M}\left(x_{jm}\left[{\left(1-\gamma_{js}e_{j}\right)}^{n_{jm}}\right]\right)$$ (21)

s.t.:

constraint (20) and

$$\sum_{j=1}^{J}\sum_{m=1}^{M}x_{jm}n_{jm}g_{j}\leq B.$$ (22)

The second strategy (problem 2 hereafter) minimizes, across all sites and all scenarios of infestation rates, the expected slippage *E*_*jms*_ when failing to detect an infestation (defined in Eq (1)), i.e.:

$$z_{2}=\min\left[\frac{1}{S}\sum_{s=1}^{S}\sum_{j=1}^{J}\sum_{m=1}^{M}x_{jm}E_{jms}\right].$$ (23)

s.t.:

constraints (20) and (22).

In practical situations, decision-makers, after undertaking a conventional delimiting survey, may decide to proceed with the removal of trees at the infested sites. We compared the efficacy of tree removal measures based on our proposed strategy and on the survey-only problems described in Eqs (21) and (23) as follows. We first solved our survey and tree removal problem for a given total budget level *B*. The solutions provided an optimal apportionment of the budget between the survey and tree removal costs. Next, we solved the survey-only problems 1 and 2 for the survey budget levels prescribed by the survey and tree removal model solutions. We then fixed the survey selection variables *x*_*jm*_ in our survey and tree removal model to the values from the model 1 and 2 solutions and re-solved the model with respect to tree removal variables *y*_1*jm*_ and *y*_2*jm*_, yielding optimal tree removal strategies for the fixed survey patterns prescribed by the survey-only problem 1 and 2 solutions.

### Minimizing the expected worst-case outcomes of survey actions

Detection errors and uncertainty about where an infestation may occur and how many trees may be infested causes the objective value to vary across the infestation scenarios *s*. The objective function in Eq (16) minimizes the *expected* number of infested trees that remain in a landscape over the distribution of all infestation scenarios. Note that the right-hand tail of that distribution contains the most severe scenarios where large numbers of infested trees in the surveyed area go undetected. In these severe scenarios, host removal or other control measures are likely to be ineffective as they are predicated on the accuracy of the survey. Knowing this, decision-makers may exercise caution and decide that the chance of missing a large number of infested trees is unacceptable and instead may try to minimize the *expected worst* outcomes of pest management actions. This behaviour is an example of ambiguity aversion [38] and widely occurs in the management of biological invasions [39–41]. When the outcomes of decision-making actions are uncertain, an ambiguity-averse manager evaluates potential actions in terms of the minimum utility that might emerge from each action. The manager then selects an action that, at the least, ensures the best of all other worst possible outcomes.

The right tail of the scenario-based distribution can be controlled with a percentile-based metric that characterises the expected tail value, such as maximum loss [42, 43], value-at-risk or conditional tail expectation. In particular, conditional value-at-risk is widely used in finance to quantify the risk of extreme losses [44–47]. In our pest management problem, value-at-risk (VaR_α_) is defined, with a confidence level *α*, *α ϵ* [0;1], as the objective function value that is exceeded in (1 –*α*)×100% of the scenarios. For a random variable, the conditional value-at-risk (CVaR_*α*_), with a confidence level *α*, is the conditional mean of the objective function values exceeding VaR_α_. For this analysis, we use CVaR_α_ to depict the ambiguity-averse strategy of minimizing the *expected worst-case* outcome of tree removal measures, i.e.:

$$min\left[CVAR_{\alpha }\left(number\;of\;infested\;trees\right)\right].$$ (24)

Since the objective function (16) in our problem was linear with respect to decision variables *x*_*jm*_, *y*_1*jm*_ and *y*_2*jm*_, we applied a linear formulation of the CVaR minimization from [46, 47] (see S1 Appendix). We composed the problem in the GAMS environment [48] and solved it with the GUROBI linear programming solver [49].

### Case study: Managing the emerald ash borer (EAB) infestation in Winnipeg, Canada

We applied our model to develop optimal strategies to manage EAB in Winnipeg, Manitoba, Canada. We used the same study area, data and assumptions as described in [30]. Detecting new EAB infestations is difficult because the infested trees stay asymptomatic for two to three years, or longer [50]. This means that the first detection of the insect in a location usually indicates that other trees are infested and will eventually require some kind of management activity (i.e., removal). Branch sampling to inspect for EAB galleries is the most reliable method to detect the insect, in particular during early stages of an outbreak when trees are otherwise asymptomatic [50, 51]. Another common method to detect EAB is to hang traps in ash trees. These traps are baited with a plant volatile and (in Canada) the EAB pheromone [52]. Trapping is less expensive on a per-tree basis, but yields lower detection rates than branch sampling [53].

**Fig 1:**
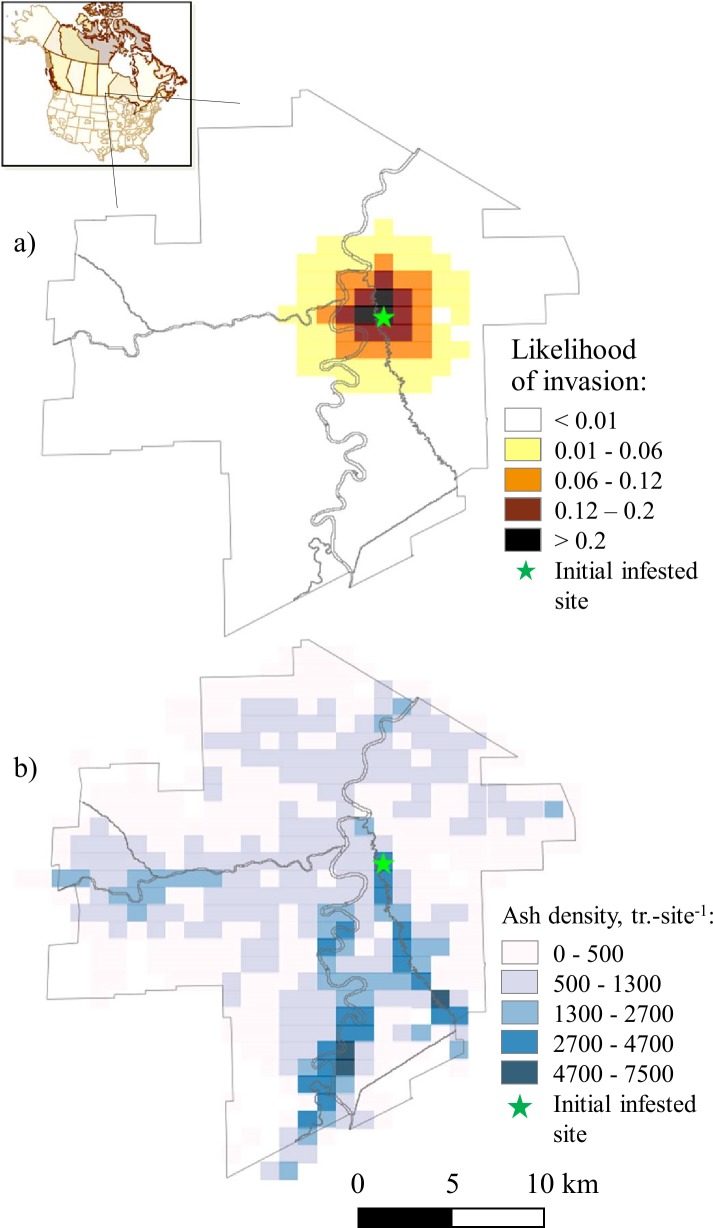
**Likelihood of infestation and ash host densities in the study area**: a) likelihood of EAB infestation (expected value based on 2000 infestation scenarios); b) ash host density, trees-km^-2^.

After the initial discovery of EAB in December 2017 [31] (Fig 1A), the City of Winnipeg and the Province of Manitoba established a program to delimit the extent of the EAB infestation in the city and remove the detected infested trees. The city was divided into a grid of 1×1 km sites. Note that the use of a 1-km survey grid ignores information about the spatial positions of the infested tree(s) within a grid cell. This may lead to coarser estimation of the infestation probabilities and could potentially affect the modelling results if the size of the grid cell is comparable with the annual spread rate of a pest. This was not the case for EAB, which has an annual spread rate exceeding 5 km, so the use of a 1-km survey grid was justified.

Some sites were inspected using the branch sampling during the winter months immediately following the initial detection, while additional sites were sampled using traps in the summer of 2018. The branch sampling method was used in the neighborhood surrounding the initial detection, while the traps were deployed in the surrounding neighborhoods and more widely within the city limits. Below we briefly describe the parameters used in our optimization model.

### Likelihoods of EAB infestation

We estimated the likelihoods of EAB arrival in uninvaded sites as a function of distance from the nearest known infested site. Modelling distance-dependent spread is a practical way to predict the likelihoods of spread for EAB [34, 54]. Because EAB was discovered only recently, we had little information about the spread of EAB in Winnipeg, so we estimated the likelihoods of introduction over distance using historical observations of the closest urban EAB infestation to Winnipeg in Minneapolis—St. Paul (Twin Cities), Minnesota [55, 56]. Evidence suggested that the pest entered the Winnipeg area six years ago, hence we used records of EAB from the Twin Cities for a six-year period starting from the oldest detection.

The spread of EAB in the Twin Cities was depicted as a map of infested trees, each with an age of infestation. For each infested tree, we estimated the distance to the group of trees with the oldest recorded infestation. We also identified the locations of other non-infested ash trees from municipal tree inventories [57, 58] and urban tree data for St. Paul used in Koch et al. [59]. We divided the area into a grid of 1×1 km sites, and for each site, estimated the proportion of infested ash trees. Next, using the EAB detection rate values collected during previous survey campaigns in the area [56], we estimated the likelihood of EAB infestation in that site. We then grouped the sites into 1-km distance classes from the infestation center and, for each distance class, estimated the distributions of likelihoods of EAB infestation.

We used the distance-dependent distributions from the Twin Cities to generate the infestation scenarios in Winnipeg. We divided Winnipeg into a grid of 1×1-km potential survey sites and for each site estimated the distance to the nearest infested area. We then sampled the distribution of infestation likelihood values for that distance from the Twin Cities data to generate the likelihood of infestation in a particular scenario. We generated 2000 infestation scenarios, which depict plausible outcomes of EAB invasion in the area. For each site, we also estimated mean likelihoods of infestation from the 2000-scenario set (Fig 1A), which depict a hypothetical single-scenario example if the manager perceives the likelihoods of EAB infestation as certain. We compared the problem solutions for the single-scenario and the 2000-scenario cases in order to see the effect of uncertainty on the results.

### Survey costs, detection rates and host densities

We estimated the ash density at each survey site from a municipal tree inventory [60] (H. Daudet, City of Winnipeg, Urban For. Br., pers. comm.), which provided information about tree species, ownership and size (Fig 1B). We used two tree size classes, 20–60 and >60 cm diameter at breast height (dbh), to adjust the cost of tree surveys because Winnipeg’s pest survey protocols only target trees with dbh >20 cm. We further assumed that inspecting trees > 60 cm dbh would require twice the effort to achieve the same detection rate as when inspecting trees 20–60 cm dbh.

The Winnipeg EAB population is thought to have been established for at least six years prior to its initial discovery, so we assumed sites where EAB was detected would contain a mix of asymptomatic and symptomatic trees. In our case, branch sampling was able to detect the presence of infestation in asymptomatic trees, so the detection rate was higher than when using traps. Based on evidence from previous surveys in Canada [50,51,53,61], we determined the efficacy of branch sampling and using traps to detect the presence of EAB. The detection rate for branch sampling was set to 0.7, based on a typical sample of two mid-crown branches from a medium-sized tree [53]. The likelihood of a single trap detecting the presence of an EAB was set to 0.5. The detection rates were determined for urban EAB populations in southern Ontario, Canada but should be applicable for Winnipeg given that its tree size distribution was typical of other urban areas in Canada.

In Winnipeg, only public trees can be inspected for EAB due to legal constraints, so we assumed that surveys would target public trees only. We calculated the survey costs using the rates paid to contractors to do branch sampling and trapping in recent EAB surveys in Winnipeg (Cdn $25-hr^-1^). We identified three broad classes of trees eligible for inspections: medium-sized accessible public (street) trees 20–60 cm dbh, large-sized accessible public trees >60 cm dbh and public woodlot, park and riparian zone trees >20 cm dbh. Inspecting trees between 20 and 60 cm dbh would require installing either one trap or sampling two branches. We assumed that inspecting trees larger than 60 cm dbh would require installing two traps or sampling four branches to achieve the same detection rate (i.e., twice the inspection effort for smaller trees). Usually, it takes longer for a surveyor to access a woodlot tree, so we doubled the costs for the site access and trap setup for woodlot trees.

Based on the constraints listed above, we estimated the total trapping cost at Cdn $87.21 for 20–60 cm dbh trees and $124.42 for trees > 60 cm dbh. This includes the cost of the trap (Cdn $24.71 and the cost of the inspection procedures of three, 15-min visits by a crew of two (for setup, sampling and teardown). Site access costs account for an additional 10 min per visit by the two-person crew.

We estimated the total cost of branch sampling at Cdn $128.90 for a 20–60 cm dbh tree and Cdn $249.60 for trees >60 cm dbh. Unlike trapping, branch sampling requires only one site visit but the total cost includes the site access by the crew of two (10-min), sampling, bark peeling and branch disposal. Sampling costs were based on estimates from the current survey campaign in Winnipeg (i.e., Cdn $65 for a 20–60 dbh tree; Cdn $121.42 for >60 cm dbh, including the site access cost). We estimated that peeling the bark from sampled branches would take 1.11 person-hours per branch and cost Cdn $55.60 for a 20–60 cm dbh tree and Cdn $111.20 for a tree > 60 cm dbh. Branch disposal included chipping the material and was expected to take 5 min for the two-person crew (Cdn $4.17 branch^-1^).

## Results

### Impact of uncertainty on optimal survey patterns

**Fig 2:**
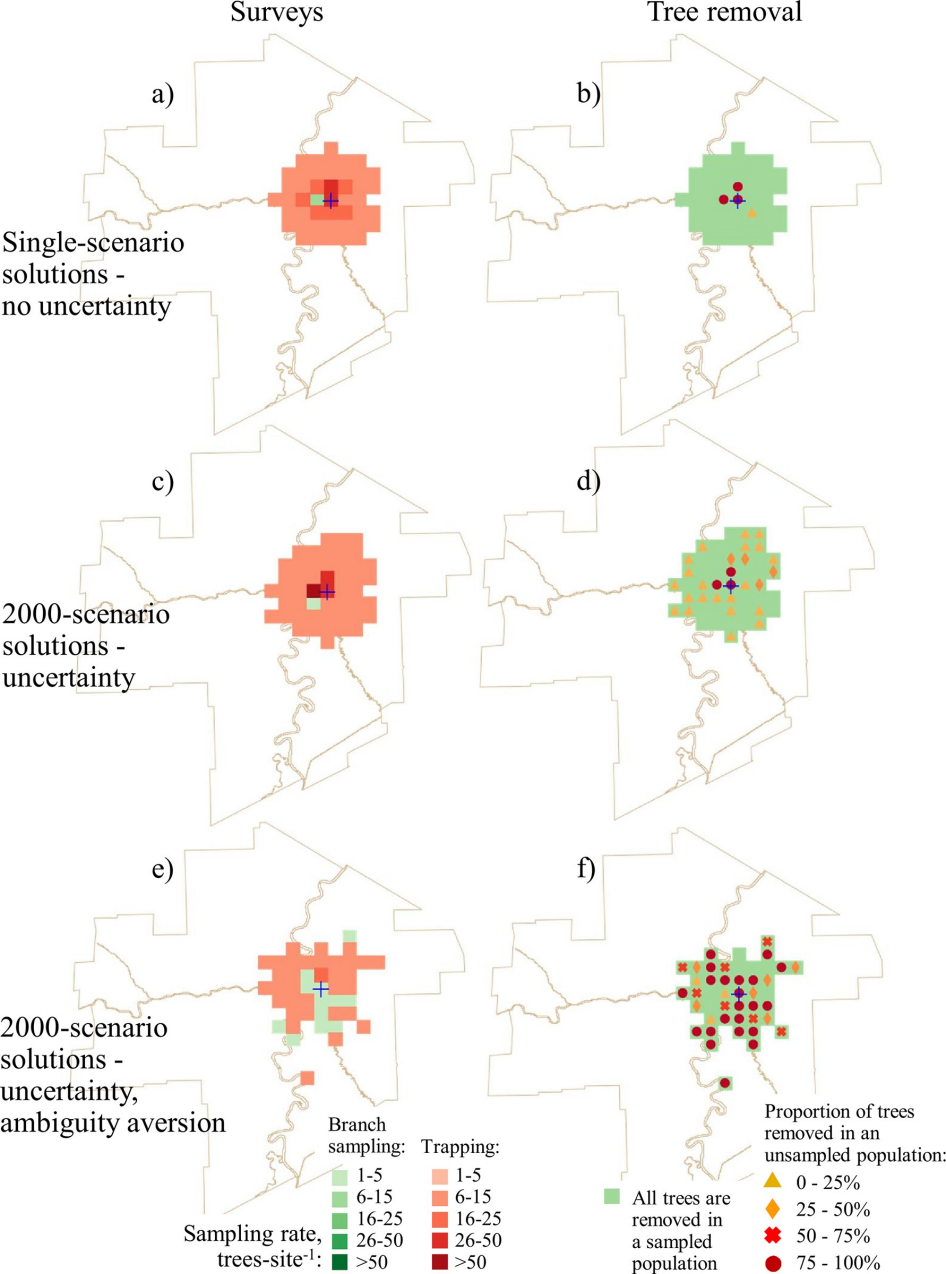
Optimal survey and tree removal patterns for a $0.8M project budget. No-uncertainty solutions: a) survey allocation; b) optimal tree removal pattern. The uncertainty solutions: c) survey allocation; d) optimal tree removal pattern. The uncertainty solutions with the ambiguity aversion assumption: e) survey allocation; f) optimal tree removal pattern.

**Fig 3:**
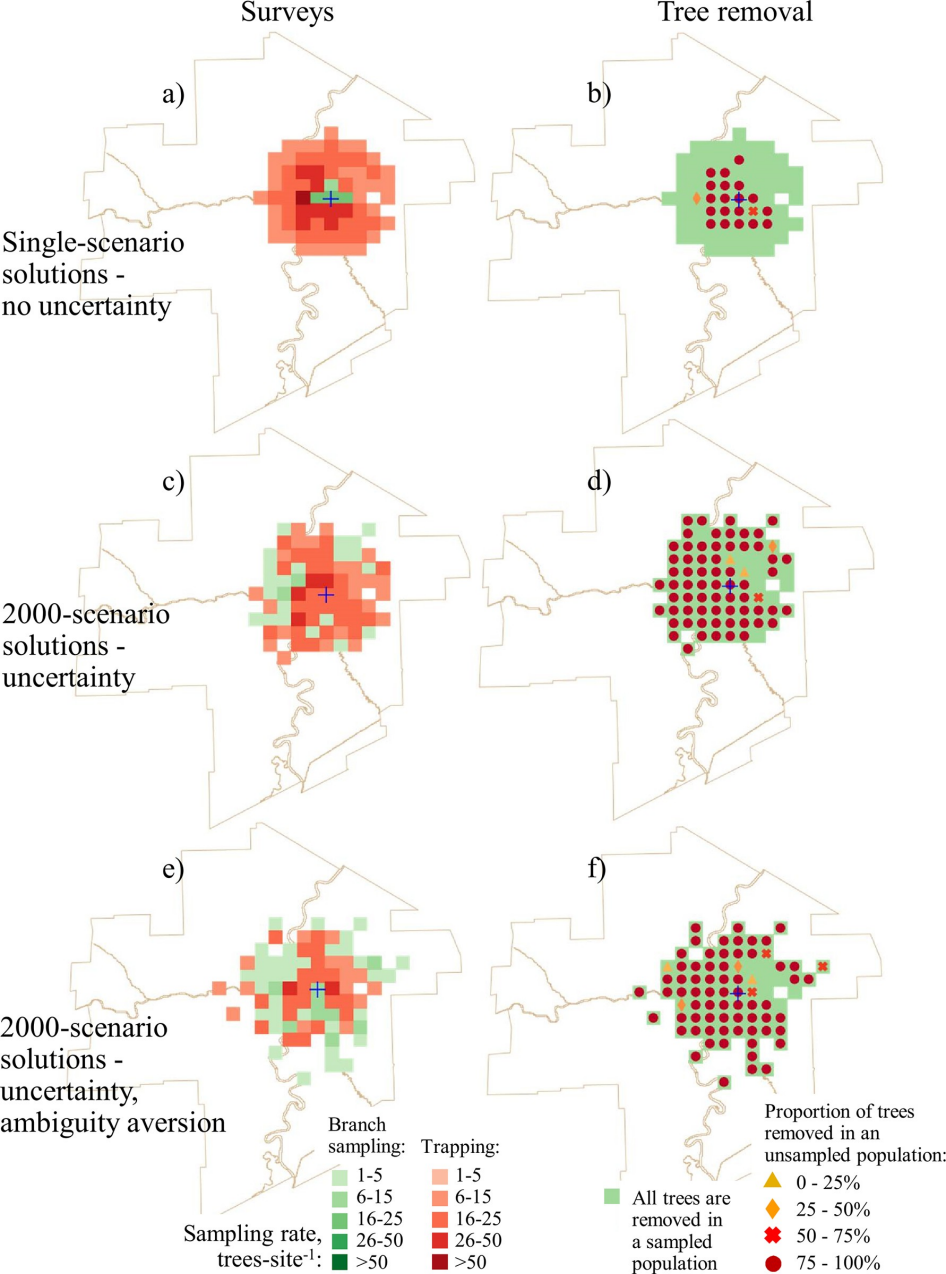
Optimal survey and tree removal patterns for a $4M project budget. No-uncertainty solutions: a) survey allocation; b) optimal tree removal pattern. The uncertainty solutions: c) survey allocation; d) optimal tree removal pattern. The uncertainty solutions with the ambiguity aversion assumption: e) survey allocation; f) optimal tree removal pattern.

We compared the optimal solutions between single-scenario formulations that used mean likelihoods of infestation and the 2000-scenario formulations (Figs 2 and 3). In all solutions, the proportion of the budget spent on surveys is very small (Table 2) because tree removal is considerably more expensive on a per tree basis than tree surveys. Recalling that the single-scenario solutions assumes that the manager knows the likelihood of EAB infestation for a particular site whereas the 2000-scenario solutions assumes that the manager only knows the approximate range of infestation likelihoods. We found that in the 2000-scenario solutions that also accounted for uncertainty (Figs 2C, 2D, 3C and 3D), more sites had trees removed after detections than in the single-scenario solutions (Figs 2A, 2B, 3A and 3B). In single-scenario solutions, branch sampling was used to inspect sites adjacent to the known infested area (which is very small) and traps were used for inspecting the rest of the city. In the solutions where the manager is ambiguity-averse, more sites were inspected via branch sampling (Fig 2E and 2F). In solutions where the available budget was large (i.e., Cdn $3M and above; Fig 3), sites proximal to the known infested area were inspected using higher sampling rates and a larger total area was surveyed than when the budget was small (Fig 2). This result shows that when a manager is ambiguity-averse, branch sampling is used more often, more sites are inspected, and more remote sites (i.e., sites that are not proximate to the known infested area) are inspected. These remote sites are primarily in parks and river valleys where the host density is high.

**Table 2:** Number of surveyed 1×1-km sites and proportions of the budget allocated to branch sampling and trapping at different survey sampling rates.

Uncertainty assumptions:	1 scenario, deterministic				2000 scenarios, uncertainty				2000 scenarios, uncertainty, ambiguity aversion			
Sampling rate	Number of surveyed sites		Allocated budget proportion		Number of surveyed sites		Allocated budget proportion		Number of surveyed sites		Allocated budget proportion	
	Br.sampl^a^.	Trap.	Br.sampl.	Trap.	Br.sampl.	Trap.	Br.sampl.	Trap.	Br.sampl.	Trap.	Br.sampl.	Trap.
**Budget limit $0.8M**												
1–5 tr.-site^-1^	-	37	-	44.4%	1	46	0.7%	34.5%	12	32	46.2%	45%
6–25 tr.-site^-1^	1	6	15.3%	14.8%	-	-	-	-	-	1	-	8.8%
26–50 tr.-site^-1^	-	2	-	25.5%	-	2	-	37.7%	-	-	-	-
51–100 tr.-site^-1^	-	-	-	-	-	1	-	27.1%	-	-	-	-
Total sites surveyed	1	45	**15.3%**	**84.7%**	1	49	**0.7%**	**99.3%**	12	33	**46.2%**	**53.8%**
Total trees surveyed	25	200			1	216			25	43		
Budget proportion spent on surveys	-	-	2.6%		-	-	3%		-	-	1.4%	
**Budget limit $1.5M**												
1–5 tr.-site^-1^	-	35	-	32.3%	19	35	19.6%	33.8%	27	26	43.6%	25.4%
6–25 tr.-site^-1^	2	8	22.6%	35.3%	-	6	-	32.6%	3	1	19.3%	11.7%
26–50 tr.-site^-1^	-	1	-	9.8%	-	1	-	14%	-	-	-	-
51–100 tr.-site^-1^	-	-	-	-	-	-	-	-	-	-	-	-
Total sites surveyed	2	44	**22.6%**	**77.4%**	19	42	**19.6%**	**80.4%**	30	27	**62.9%**	**37.1%**
Total trees surveyed	48	231			26	156			62	54		
Budget proportion spent on surveys	-	-	1.8%		-	-	1.6%		-	-	1.3%	
**Budget limit $4M**												
1–5 tr.-site^-1^	-	32	-	10.6%	21	23	11.9%	9.4%	34	15	19.7%	6.2%
6–25 tr.-site^-1^	1	26	3.9%	27.7%	2	28	2.6%	56.1%	9	20	15.3%	41.6%
26–50 tr.-site^-1^	3	9	15.8%	35.8%	-	4	-	19.8%	-	2	-	17.2%
51–100 tr.-site^-1^	-	1	-	6.2%	-	-	-	-	-	-	-	-
Total sites surveyed	4	68	**19.7%**	**80.3%**	23	55	**14.5%**	**85.5%**	43	37	**35%**	**65%**
Total trees surveyed	112	742			67	556			156	384		
Budget proportion spent on surveys	-	-	2.1%		-	-	2%		-	-	2%	

^a^ Survey methods: Br.sampl.–branch sampling, Trap.–trapping.

In single-scenario solutions, the removal of the unsampled tree population after detection was only recommended for sites in close proximity to the known infested area (Figs 2B and 3B). In the 2000-scenario solutions, adding uncertainty resulted in prescriptions for the removal of some unsampled trees after detection across nearly all of the surveyed area (Figs 2D and 3D). In the solutions where the manager is ambiguity-averse, the prescription was for a larger proportion of the unsampled tree population to be removed, and more trees are removed from remote sites (Figs 2F and 3F).

In large-budget solutions, the optimal tree removal strategy after detection was to remove all trees in both the sampled and unsampled populations except in sites with very low host densities. At low density sites the prescription was only to remove the sampled trees. In short, complete tree removal after detection is a strategy to compensate for uncertainty. This is because the actual infestation rate in the survey area is unknown, therefore it is impossible to stratify the removal of the unsampled trees. Thus, the optimal strategy is to remove all trees at a site after detection. Though, the extent of tree removal across a city is still limited by the project budget.

### Optimal apportionment between survey and tree removal budgets

Without uncertainty, in the single-scenario solutions, trapping was the most used inspection method, with its share of the coverage of the surveyed area ranging from 77 to 85% (Table 2). Adding uncertainty in the 2000-scenario solutions makes the choice of the inspection method more dependent on the project budget. In small-budget solutions, most of the area was surveyed using traps. This is unsurprising because a larger area needs to be surveyed in order to compensate for the uncertainty and the only way to achieve this with limited survey funds is to use the cheaper trapping method. However, when the budget is large, more funds can be set aside for surveys and more sites are inspected with the more reliable branch sampling method (Table 2).

When we assumed the manager is ambiguity-averse, this further increased the proportion of sites surveyed via branch sampling, but also made the choice of the inspection method less dependent on the budget. Being averse to ambiguity necessitates inspection of more remote sites in areas of high host density, where failed inspections could lead to significant damage to the host resource.

With respect to tree removal, single-scenario solutions prescribed the removal of the largest number of trees and the uncertainty scenarios with ambiguity prescribed the removal of the smallest number of trees (Table 3). However, removal occurred over more sites in the ambiguity-averse scenarios, and furthermore, more of the removed trees were from the unsampled tree populations. This occurred because the removal of trees is conditional on detecting the pest and yet the true infestation rate in the unsampled tree population of a site is unknown. Therefore, the manager–after a detection in the sampled population–must remove all the unsampled trees to guarantee that all of them that might be infested are removed. As noted previously, the likelihood of infestation at remote sites is lower than at sites in close proximity to the known infested area. This means that the expected proportions of infested trees in the sampled and unsampled populations of remote sites are also lower. In turn, this means the removal of unsampled trees from remote sites is less cost-effective than is removal of trees from nearby sites. Thus, the penalty for compensating for uncertainty about the ability of the invader to spread, particularly in combination with an ambiguity-averse manager (*cf*. the avoidance of worst-case damage), is to inspect remote sites, where both survey and removal are comparatively inefficient but necessary so as to minimize the chance of a major undetected expansion by the invader.

**Table 3:** Expected number of trees removed and the budget proportion spent on tree removal in sampled and unsampled populations.

Uncertainty assumptions:	1 scenario, deterministic				2000 scenarios, uncertainty				2000 scenarios, uncertainty, ambiguity aversion			
Sampling rate	Tree removal		Allocated budget proportion		Tree removal		Allocated budget proportion		Tree removal		Allocated budget proportion	
	SP^a^.	UP^b^	SP.	UP	SP.	UP	SP.	UP	SP	UP	SP.	UP
**Budget limit $800000**												
Total sites with tree removal	46	4	12.1%	87.9%	50	28	15.5%	84.5%	45	38	2.3%	97.7%
Exp. number of removed trees	118	855			127	695			14	626		
**Budget limit $1500000**												
Total sites with tree removal	46	11	9.2%	90.8%	61	52	5%	95%	57	50	2.3%	97.7%
Exp. number of removed trees	166	1673			69	1306			30	1270		
**Budget limit $4000000**												
Total sites with tree removal	72	21	12.2%	87.8%	78	69	6.8%	93.2%	80	73	5.6%	94.4%
Exp. number of removed trees	598	4298			254	3485			206	3465		

^a^ SP–sampled population;

^b^ UP–unsampled population.

### Survey allocation and the tree removal budget

In some situations, budgets for invasive species management programs can be apportioned separately between survey and management actions. We explored how varying the amount of the budget set aside for tree removal and the potential under- or over-allocation of tree removal funds may influence the optimal survey strategy. We found the solutions for a fixed survey budget and a set of different tree removal budgets. These solutions depict circumstances where a city’s survey budget and tree removal budget come from different funding sources decided on through separate planning procedures. We then compared these solutions with the solution that optimally apportioned the budget between survey and tree removal and had similar funding level allocated to surveys.

**Fig 4:**
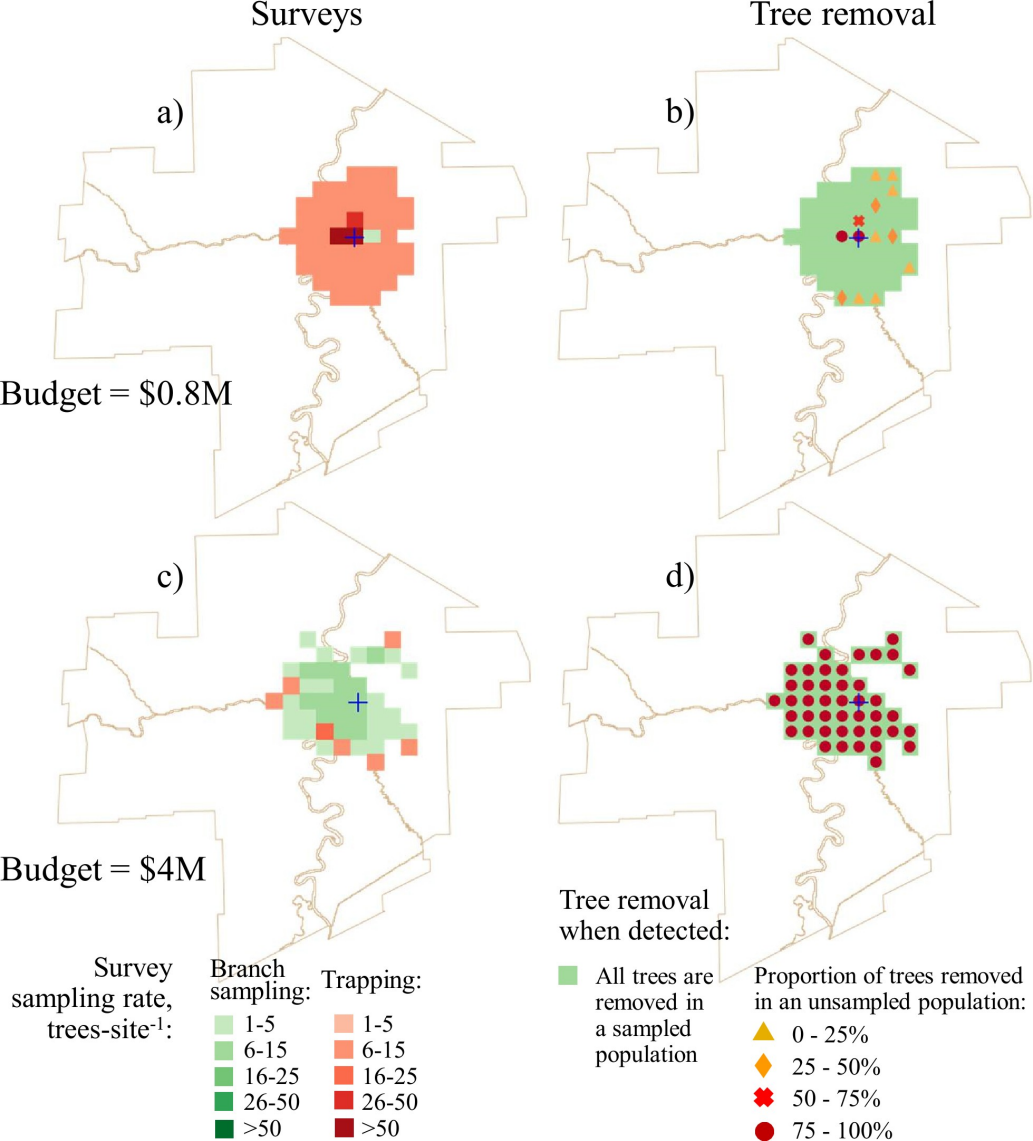
Optimal survey and tree removal patterns for the solutions with fixed survey budgets and different tree removal budgets. The survey budget $25000 and total project budget $0.8M ($0.775M spent on tree removal): a) survey allocation; b) optimal tree removal pattern. The survey budget $25000 and total project budget $4M ($3.975M spent on tree removal): c) survey allocation; d) optimal tree removal pattern.

**Fig 5:**
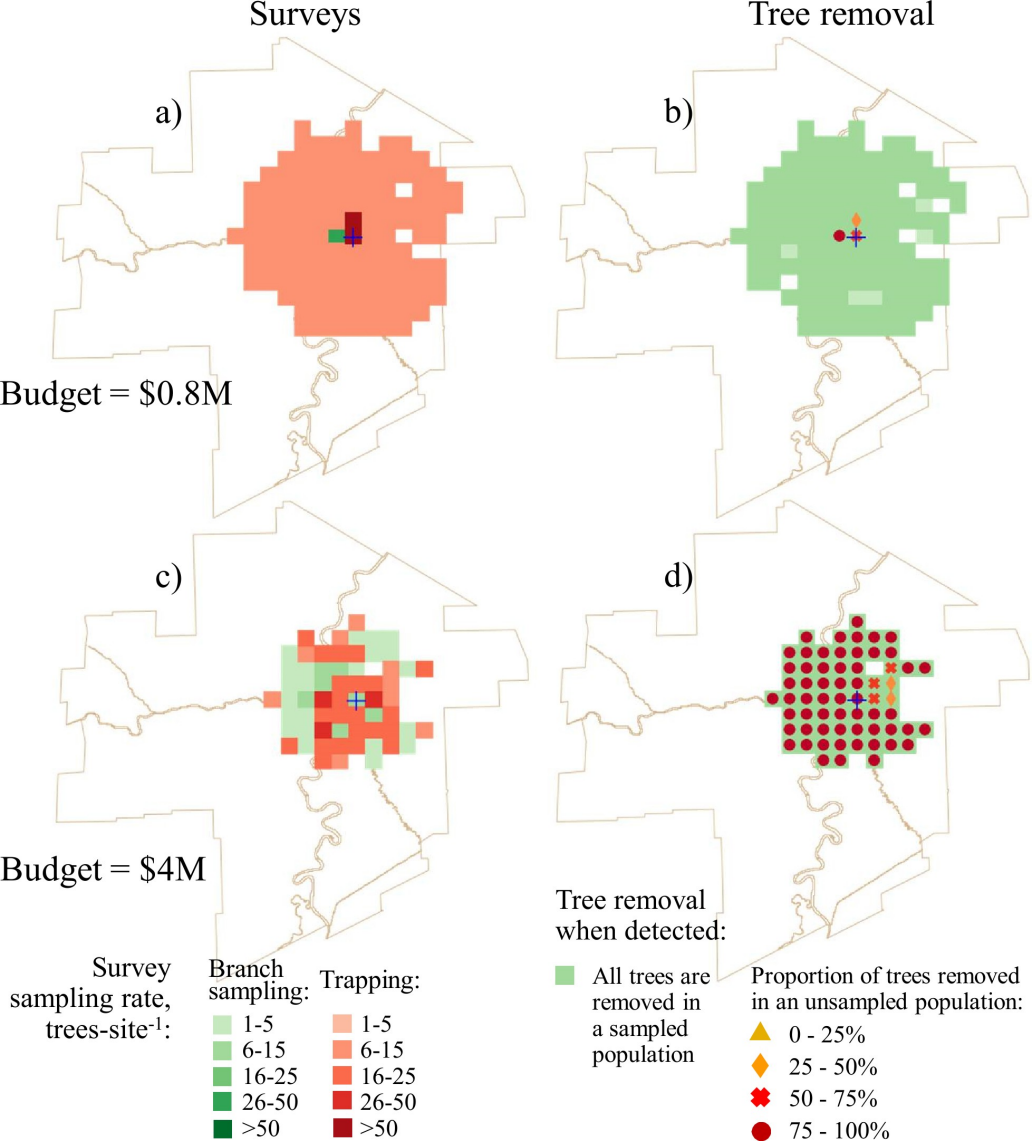
Optimal survey and tree removal patterns for the solutions with fixed survey budgets and different tree removal budgets. The survey budget $50000 and total project budget $0.8M ($0.775M spent on tree removal): a) survey allocation; b) optimal tree removal pattern. The survey budget $50000 and total project budget $4M ($3.975M spent on tree removal): c) survey allocation; d) optimal tree removal pattern.

Figs 4 and 5 show the optimal survey and tree removal solutions for the survey budgets of Cdn $25k and $50k and total project budgets Cdn $0.8M and $4M. The solutions with a total budget of $0.8M depict a situation when the manager has limited funds to remove trees (Figs 4A,4B, 5A and 5B). In these solutions, most of the area was inspected using traps at low sampling rates. High sampling rates and branch sampling were only used at sites adjacent to the known infested area. All sampled trees were removed at the sites with positive detections, but the removal of unsampled trees only occurred at a few sites adjacent to the known infested area that were subject to high sampling rates. In summary, if the tree removal budget is relatively small, high sampling rates should only be used in the sites where intensive removal of trees is expected (i.e., in close proximity to the known infested area). The rest of the survey budget should be spent on low-intensity surveys using the least expensive method.

When sufficient funding is available for tree removal, the optimal prescription is to remove all the host trees from all sites where infested trees were found. There is a trade-off however, in large-budget solutions the total surveyed area is smaller than in small-budget solutions but the surveys use higher sampling rates. The budget available for tree removal determines the total survey area assuming the removal of all trees from any site with a detection (Figs 4D and 5D).

Tree removal is most effective when the portion of the budget allocated to survey is close to its optimal value. Table 4 shows the expected number of removed infested trees for three fixed survey budgets at small ($0.8M) and large ($4M) total budget levels. In general, the fixed-survey solutions closest to the optimal apportionment of survey and tree removal costs also prescribe the removal of the highest number of trees. When the survey budget is set above its optimal value, less funding is available for tree removal. When the survey budget is too low, tree removal doesn’t work because too many infested trees are likely missed.

**Table 4:** Expected number of removed infested trees for fixed survey budgets. The uncertainty solutions with 2000 scenarios are shown.

Budget limit, $	Survey cost allocation	Survey cost, $	Expected number of removed infested trees
0.8M	**Optimal**	**18966**	**285.8**
	Fixed	25000	285.7
	Fixed	50000	279.3
	Fixed	100000	262.6
4M	Fixed	25000	872
	Fixed	50000	960.3
	**Optimal**	**59484**	**964.9**
	Fixed	100000	960.5

### Surveys with tree removal vs. the survey-only strategies

**Fig 6:**
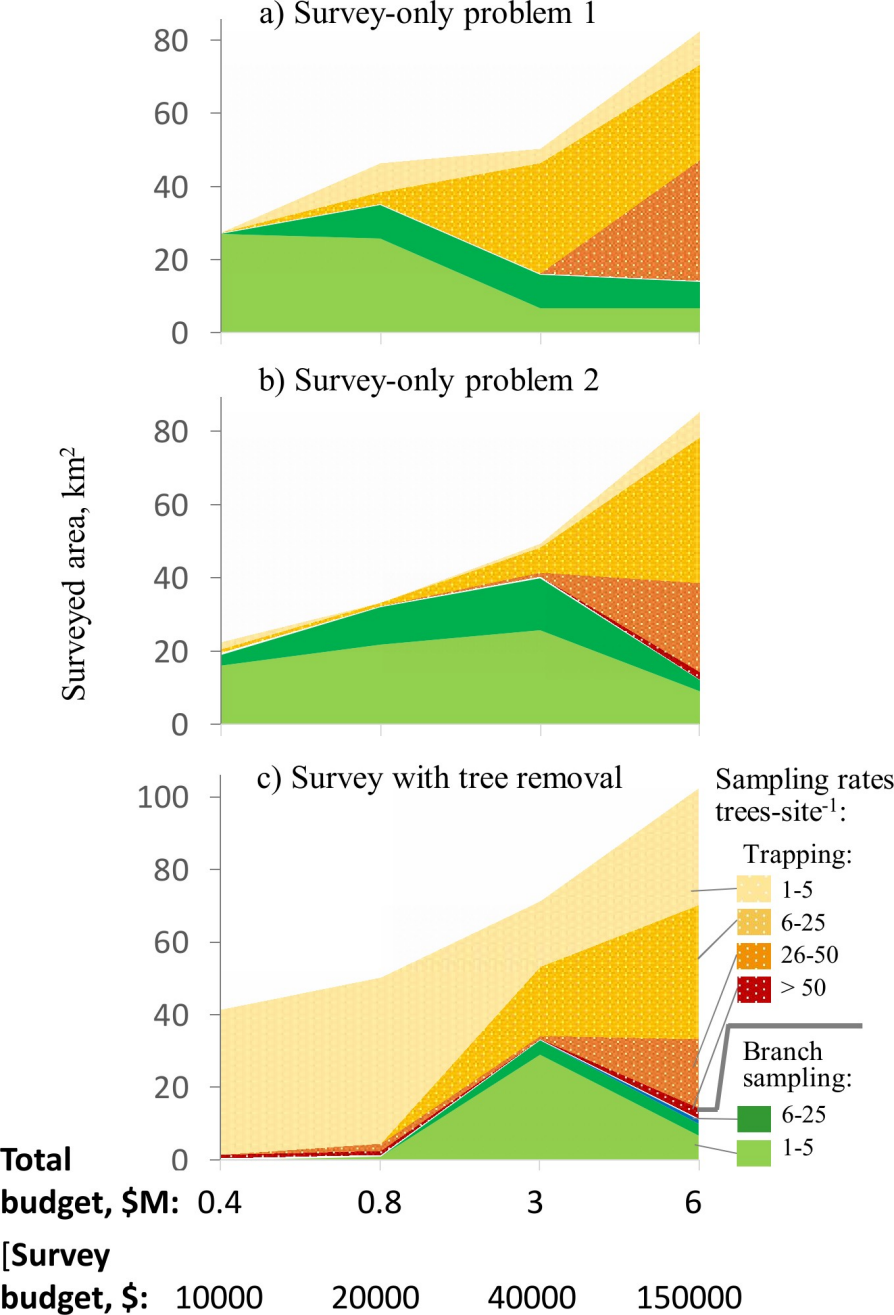
Area surveyed with different sampling rates in the solutions with tree removal and the survey-only problem 1 and 2 solutions. Colors / shades show the areas surveyed at a particular tree sampling rate and using a particular survey method. X-axis denotes the total budget, $ million, secondary X-axis shows the survey budget portion, $, and Y-axis denotes the survey area, km^2^: a) surveys based on problem 1 objective; b) surveys based on problem 2 objective; c) surveys in the optimal solution with tree removal.

**Fig 7:**
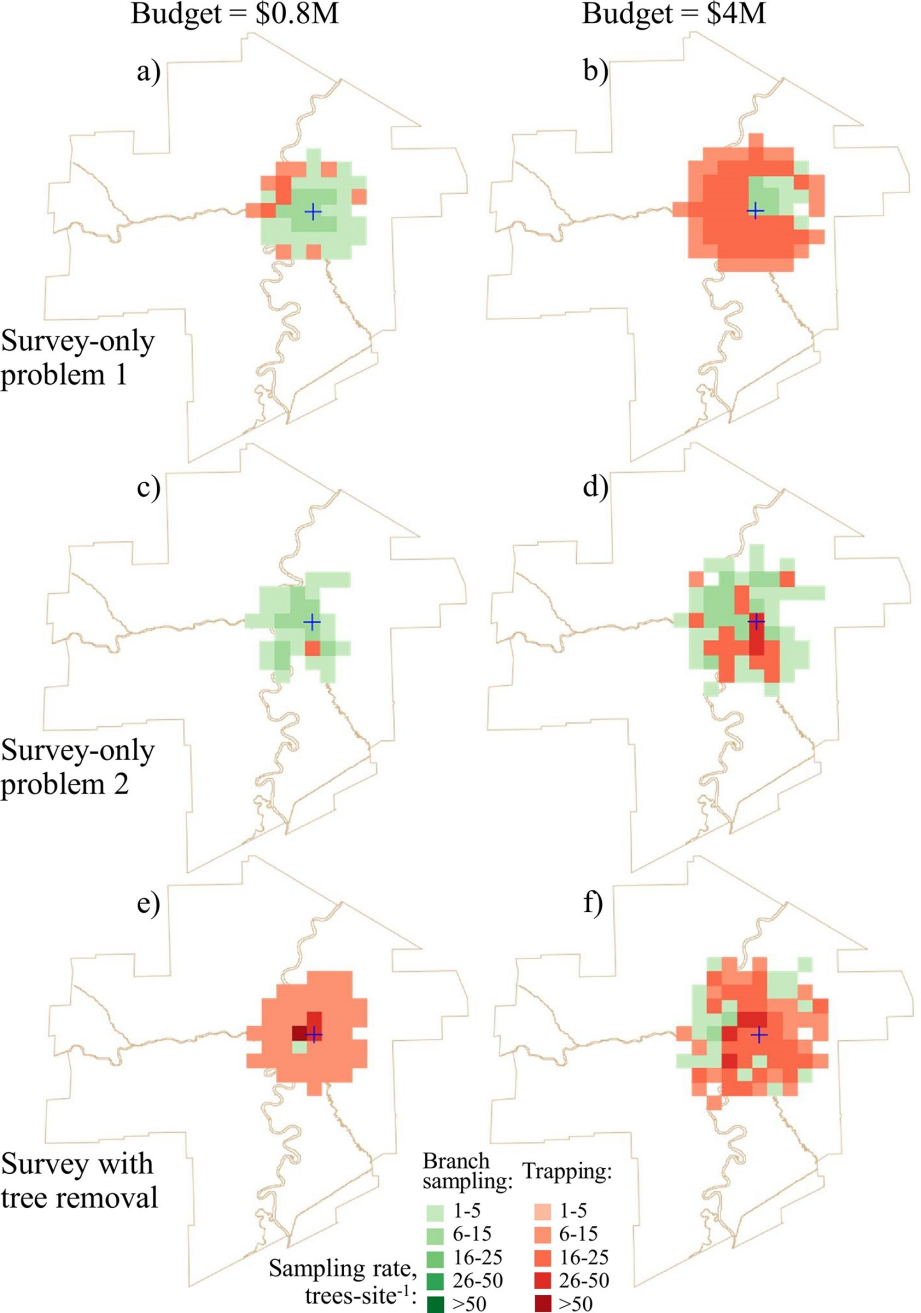
Optimal survey patterns in the solutions with tree removal and the survey-only problem 1 and 2 solutions. Project budget $0.8M: a) survey-only problem 1 solution; c) survey-only problem 2 solution; e) optimal survey solution with tree removal. Project budget $4M: b) survey-only problem 1 solution; d) survey-only problem 2 solution; f) optimal survey solution with tree removal. The survey cost portions in the $0.8M and $4M project budgets are approximately $0.019M and $0.059M.

Plans that dictate the removal of trees after detecting an infestation (survey-removal) have a different survey strategy than plans that do not anticipate management actions after detection (survey-only). We compared our optimal survey-removal solutions to survey-only strategies implemented as problems 1 and 2 described in [30] via Eqs (21) and (23), respectively. Figs 6 and 7 show pronounced differences in survey-only strategies between the problem 1 and 2 solutions and the solutions for our survey-removal approach. For small budgets (with the survey cost below $40,000), branch sampling was preferred in problem 1 and 2 solutions (Figs 6A,6B, 7A and 7C) but trapping was preferred in the survey-removal solutions (Figs 6C and 7C). The proportional use of branch sampling and trapping in the survey-only and survey-removal solutions starts to converge at large budgets (Fig 6), but the spatial patterns of where surveys should occur still show notable dissimilarities (Fig 7B,7D and 7F).

These differences in sampling strategies stem from distinct survey objectives. The survey-only and survey-removal approaches adopt different philosophies. A survey-only approach intends to get a handle on the extent of an invasion, in preparation for planning future response actions; because resources are always limiting, a decision maker wants to start taking action where the invader is a priority. This is a reactive measure. Alternatively, the survey-removal approach tries to limit the scope (and thus the expected impact) of the invasion in the immediate future by removing trees in recently detected infestation nuclei. This is a proactive measure. Compared to the survey-only solutions, the survey-removal approach does not maximize the capacity to detect infestation or minimize the number of infested trees in false negatives *per se*. Instead, the survey-removal approach attempts to minimize the expected number of infested trees remaining after removal. Recall that surveys have to compete with tree removal within the budget allocation. The smaller the tree removal budget the fewer sites where complete tree removal is cost-effective. In this case, a two-tiered strategy is optimal: survey a small number of sites at high sampling rates with a method that gives the best chances of detection and survey the rest of the area with low sampling rates with the least expensive detection method. Comparatively, the survey-only strategies prescribe higher sampling rates and use of a more reliable inspection method to maximize the number of successful detections, inspecting a smaller area as a result. When used to guide removal efforts, the survey-only strategies also lead to removal of fewer infested trees overall (Table 5).

**Table 5:** Expected number of infested trees removed in the solutions with different problem objectives. The survey-only strategies 1 and 2 use problem objectives from Eqs [21] and [23].

Survey problem	Total budget, million $			
	0.4	0.8	1.5	4
**Uncertainty scenarios:**				
**Survey with tree removal**	**144.9**	**285.8**	**475.1**	**964.9**
Survey-only problem 1	127.4	257.6	447.5	934.2
Survey-only problem 2	131.1	260.1	444	925.4
**Uncertainty scenarios, ambiguity aversion:**				
**Survey with tree removal**	**120.5**	**232.7**	**450.5**	**953.2**
Survey-only problem 1	109	230.5	443.8	932.1
Survey-only problem 2	110	220.2	402.5	915.2

### Sensitivity analysis

We estimated the sensitivities of key output metrics to changes in the model parameters (Table 6). Rows in Table 6 denote the input model parameters of interest (i.e., the survey and tree removal unit costs, the detection and infestation rates and the host density) and columns denote the output metrics of interest. The sensitivity values indicate the relative change of the output metric (columns in Table 6) in response to changing the input parameter (rows in Table 6) by ±20%. In addition to testing the objective value, we also examined the sensitivities of other relevant outputs to changes in model parameters, such as the number of sites surveyed via branch sampling and trapping, the budget portion spent on surveys and the proportions of trees removed from sampled and unsampled tree populations.

**Table 6:** Sensitivity analyses exploring the response of model outputs to varying model input parameters by +/- 20%^a^.

Model parameter	Objective value: Exp. number of remaining inf. Trees	Number of surveyed sites		Budget portion spent on surveys	% trees removed after detection	
		Via branch sampling	Via trapping		Sampled population	Unsampled population
**Total budget $0.8M:**						
Survey cost	0.02	**6.0**^**b**^	0.29	0.47	<0.01	0.14
Detection rate	0.03	**4.0**	0.49	0.22	<0.01	0.43
Host density	<0.01	2.0	0.12	0.06	<0.01	0.25
Infestation rate	**25.6**	2.0	0.16	0.62	<0.01	0.10
Tree removal cost	0.89	**8.0**	0.37	0.91	<0.01	**10.13**
**Total budget $4M:**						
Survey cost	0.01	0.43	0.36	0.88	<0.01	0.16
Detection rate	0.02	1.13	0.40	0.97	<0.01	0.51
Host density	0.00	0.17	<0.01	0.02	<0.01	0.29
Infestation rate	**3.45**	0.61	0.15	0.59	<0.01	0.26
Tree removal cost	**3.70**	2.35	1.05	0.87	<0.01	0.36

^a^ Sensitivity value 1.0 indicates that the relative change of the parameter by +/- 20% causes the change in the output values by +/-20%.

^b^ Sensitivity values 3.0 and above are in **bold**.

As expected, the model objective was most sensitive to changes in the infestation rate and moderately sensitive to changes in the unit cost of tree removal. The infestation rate defines the expected number of infested trees in a landscape. Sensitivity to changes in the infestation rate was highest in small-budget solutions because the limited funds allowed removal of only a small proportion of the infested trees, and so the expected number of remaining infested trees was essentially a function of the infestation rate.

In small-budget solutions, the budget proportion spent on surveys was moderately sensitive to the unit cost of tree removal and the infestation rate. The use of branch sampling was highly sensitive to changes in the survey and tree removal costs and, to a lesser degree, to changes in the detection rate. The use of trapping is less sensitive to changes in the model parameters because it was the more commonly used method.

Changes in the model parameters did not affect the number of sampled trees that were prescribed for removal because all solutions prescribed removal of all sampled trees. The proportion of the unsampled trees that were prescribed for removal was most sensitive to changes in the unit cost of tree removal in small-budget solutions and moderately sensitive to changes in the detection rate. The impacts of changing the detection and infestation rates were slightly more evident in large-budget solutions than in small-budget solutions, because in large-budget solutions more trees were removed across a larger area, and so the ability to detect the infestation becomes more critical.

## Discussion

Planning a response to a biological invasion is a balancing act of distributing scarce resources between surveillance and control. Managers often have limited understanding about how a pest might spread through their area of concern, which may hinder their decisions on where to apply costly control measures. Our approach helps address this challenge and demonstrates how accounting for the uncertainty and decision-makers’ perceptions of it may influence control decisions. Decision-makers always face the risk that wrong assumptions lead them towards incorrect decisions. Our results demonstrate that omitting this uncertainty may push managers to survey and initiate tree removal in a smaller area than may be advisable. Accounting for uncertainty prompts survey of a larger area and, when the budget permits, spending the funds on the removal of all sampled and unsampled trees across all sites with positive detections.

Our results also provide new insights about the utility of using trapping versus branch sampling techniques for EAB detection. When a community’s project budget is small and their survey aims to guide tree removal efforts, branch sampling is only advisable in sites with a high probability of successful detections and where tree removal is most likely to occur. The rest of the area should be inspected with the cheaper trapping method to preserve more funds for tree removal. The use of branch sampling is only advised for widespread use in communities where the budget is big enough to also undertake a large-scale tree removal effort (although trapping still remains the predominant inspection method).

The application of branch sampling and trapping in our survey-removal solutions was distinct from how they were applied in the survey-only strategies. At small budgets, traps were used to inspect most of the area in our survey-removal strategies (Fig 7C) whereas branch sampling predominated in the survey-only strategies (Figs 6, 7A and 7B). Under our approach, branch sampling was more effective for inspecting sites proximal to the known infested area or sites with low host densities (so the inspections of fewer trees could lead to a detection). The use of the cheaper trapping method elsewhere helps maximize the survey area and ensures that enough detections can be made to allocate the tree removal budget.

### Impact of the uncertainty and ambiguity-averse perceptions on tree removal strategies

Uncertainty about an invader’s spread changes the tree removal strategy for the unsampled trees in a survey site. Without uncertainty, our tree removal solutions prescribe the removal of unsampled trees after detection only in sites that also have a high probability of pest introduction, because the prescriptions tended to follow the spatial pattern of where infestations were likely. When information about an invader’s spread is acknowledged as uncertain, the optimal strategy changes to one where, after detection, a manager should remove all trees in the sampled and unsampled populations. Thus, tree removal becomes more spatially uniform to compensate for uncertainty. This behaviour also persists when the manager is ambiguity-averse and striving to avoid the worst-case outcome of having large numbers of infested trees remaining in the area. Ambiguity-averse solutions usually prescribe surveys and tree removal in remote sites with high host densities, where the pest is less likely to spread, but if gone undetected, could cause serious damage.

### Tree removal vs. survey-only strategies

Factoring potential host removal directly into the overall response strategy makes the removal of infested trees more cost-effective. Our results show distinct differences between survey-only and survey-removal solutions, particularly at small budgets (Fig 7A, 7C and 7E). This highlights a fundamental difference in the survey objective when host removal will be the response to a detection. Finding the full extent of the infestation *per se* is no longer the main goal of the survey. Instead, inspections become the means by which a manager can reduce the number of infested trees in a landscape and so needs to ensure a sufficient number of detections to effectively spend the tree removal budget. Because tree removal is expensive, any savings in the survey budget can be spent to remove more infested trees from the area of concern. However, if the manager anticipates that the tree removal funds will be insufficient then a two-tiered strategy should be adopted. High sampling rates should only be applied to sites proximal to the known infested area, where arrival of the pest and the subsequent removal of trees is most likely, and the rest of the area should be inspected using low sampling rates and the least expensive inspection method.

### Future work

Our case study followed the current surveillance practices adopted by the city of Winnipeg where surveys and tree removal only affect public trees. It is possible that including private trees could change the tree removal prescriptions. However, evidence from previous tree removal campaigns in southern Ontario suggests that inspecting and removing trees on private property in an urban setting is often more expensive than removing public trees due to access and liability constraints. Thus, when a budget only allows inspecting and removing a small number of trees, the most cost-effective strategy would still be to inspect public street trees only.

Dealing with uncertainty about an invader’s pattern and rate of spread is a familiar challenge for pest management professionals. Precise estimates of spread rates and likelihoods of pest introductions are rarely available for newly detected infestations and can only be approximated from previous infestations or from knowledge of the organism’s ecology. Our results highlight the importance of proper estimation of the expected spread rates for new infestations. In our study, we estimated the likelihoods of EAB spread from recent infestation in the Twin Cities. While the spread of this EAB infestation has been well documented and good ash density data area available, the area also has a warmer climate than Winnipeg which could have lead to an overestimate of the spread rate. For instance, colder winters in Winnipeg may cause EAB to switch to a longer, two-year life cycle, which would result in both lower population growth rate and a slower spread rate. However, given that long-distance dispersal of EAB is often a result of human activities, the impact of colder climate on spread of EAB within a city may be difficult to estimate. Calibrating the long-distance spread assumptions would require better understanding of the EAB’s ecology in Winnipeg and will be a focus of future work.

Another potential model enhancement could be adding a chemical treatment option for host trees that are found to be infested. Most insecticides available for control of EAB in the United States are available for purchase in Canada, but have not been registered for use against the insect due to various reasons: environmental and legal restrictions, stringent requirements in terms of application methods and generally small markets (i.e., less demand) for pesticides to treat urban trees). In Canada, there are presently two products available, TreeAzin (azadiractin) and ImaJet (imidacloprid), for treating ash trees via trunk injections. TreeAzin is the more commonly used product and is marketed as effective for two years after application. For Canada, the manufacturer of ImaJet (Arborjet) suggests reapplication every year. Using either of these products to treat infested ash trees in Winnipeg is less cost-effective than treating equivalent trees in the eastern U.S., where less expensive and longer-lasting options are registered. This is why we focused on the tree removal option in this case study. Potentially, our model could be adapted to include both chemical treatment and tree removal options. This requires switching from a one-stage to a multi-stage problem formulation to account for the temporary effect of treatment actions, and will be the focus of future efforts.

## Supporting information

S1 Appendix Minimizing the expected worst-case outcome of tree survey and removal measures. (DOC)
