# Supplementary material for: Managing biological invasions in urban environments with the acceptance sampling approach
Source: PLoS One. 2019 Aug 23;14(8):e0220687. doi: 10.1371/journal.pone.0220687 (PMC6707552; doi:10.1371/journal.pone.0220687)
Supplement: S1 Appendix — (DOC) [file pone.0220687.s001.doc]

**Supporting information**

**S1. Minimizing the expected worst-case outcome of tree survey and removal measures**

Minimizing the expected worst-case detection time requires controlling the values in the right tail of the detection time distribution. This can be achieved using an upper-percentile-based metric, such as Conditional Tail Expectation (CTE), also known as Conditional Value-at-Risk (CVaR). In our case, CVaR for a confidence level *α*, *α* ∈ [0;1], can be defined as the expected time to first detection over the (1 – *α*)×100% worst scenarios, i.e., the (1 – *α*)×100% of scenarios with the longest times to first detection (Fig. S1.1). For a random variable, CVaR_α_ is the conditional mean of the values (i.e., times to first detection) exceeding VaR_α_, where VaR is the value in the distribution of detection times in *S* scenarios that is exceeded only in the (1–*α*)×100% worst scenarios with longest detection times.

**Fig.S1.1. The concept of minimizing the expected number and CVaR of the number of infested trees after survey and tree removal measures.**

When the objective function is linear with respect to the decision variables, the conditional value at risk (CVaR) can be incorporated in an optimization framework [1-3]. For discrete scenario-based distributions, optimizing CVaR with respect to linear decision variables can be expressed by a set of linear equations [2,3]. In our case, the objective function is linear with respect to decision variables *x_jm_*, *y*_1_*_jm_* and *y*_2_*_jm_*, i.e.:

 $z=\min\frac{1}{S}\sum_{s=1}^{S} \sum_{j=1}^{J} \sum_{m=1}^{M} \left[x_{jm}\gamma_{js}P_{jms}(N_{j}-n_{jm}[1-\beta_{js}])+\gamma_{js}[1-P_{jms}](N_{j}-n_{jm})(x_{jm}-y_{2jm})+\gamma_{js}n_{jm}[1-P_{jms}\beta_{js}](x_{jm}-y_{1jm})\right]$ (S1.1)

(see Equation (16) and symbol definitions in Table 1 in the main text). The objective can be modified to minimize the CVaR_α_ of time to first detection using the formulation from Rockafellar and Uryasev [2,3]. For a discrete distribution of *S* invasion scenarios with a probability of occurrence 1/*S*, minimization of the CVaR*_α_* can be approximated with the following objective function formulation and equivalent sets of *S* + 1 auxiliary decision variables and *S* + 1 inequality constraints:

 $\min[\zeta+\frac{1}{S(1-\alpha)}\sum_{s=1}^{S} w_{s}]$ (S1.2)

s.t.:
 $w_{s}\geq\sum_{j=1}^{J} \sum_{m=1}^{M} \left(x_{jm}\gamma_{js}P_{jms}(N_{j}-n_{jm}[1-\beta_{js}])+\gamma_{js}[1-P_{jms}](N_{j}-n_{jm})(x_{jm}-y_{2jm})+\gamma_{js}n_{jm}[1-P_{jms}\beta_{js}](x_{jm}-y_{1jm})\right)-\zeta$  *s* ∈ *S* (S1.3)
 *w_s_* ≥ 0  *s* ∈ *S*  (S1.4)

and the constraint equations (1), (6), (9), and (10) in the main text,
where
$\sum_{j=1}^{J} \sum_{m=1}^{M} \left(x_{jm}\gamma_{js}P_{jms}(N_{j}-n_{jm}[1-\beta_{js}])+\gamma_{js}[1-P_{jms}](N_{j}-n_{jm})(x_{jm}-y_{2jm})+\gamma_{js}n_{jm}[1-P_{jms}\beta_{js}](x_{jm}-y_{1jm})\right)$is the number of infested trees in a site *j* where the inspection of *n_jm_* trees did not find an infestation or no survey occurred, plus the expected number of infested trees after the inspection of *n_jm_* trees has found one or more infested trees and tree removal may have occurred in a scenario *s*, *ζ* and *w_s_* are auxiliary decision variables and *ζ* is a member of a set of real numbers. In this formulation, the survey allocation follows an ambiguity-averse strategy by minimizing expected worst outcomes, i.e., expected maximum number of infested trees in the area after the survey and tree removal efforts. We set the confidence level *α* in CVaR equations was set to 0.95, which defines a conditional expectation of the distribution tail above the 95^th^ percentile.

**References:**

1. Acerbi C, Tasche D. Expected shortfall: a natural coherent alternative to Value at Risk. Economic Notes 2002; 31(2): 379-388.

2. Rockafellar RT, Uryasev S. Optimization of Conditional Value-at-Risk. Journal of Risk 2000; 2: 21-41.

3. Rockafellar RT, Uryasev S. Conditional value-at-risk for general loss distributions, Journal of Banking & Finance 2002; 26: 1443-1471.
